# Who do phone surveys miss, and how to reduce exclusion: recommendations from phone surveys in nine Indian states

**DOI:** 10.1136/bmjgh-2021-005610

**Published:** 2021-08-11

**Authors:** Karan Nagpal, Mitali Roy Mathur, Abhilash Biswas, Andrew Fraker

**Affiliations:** 1 ID Insight India Private Limited, New Delhi, India; 2 IDinsight Inc, San Francisco, California, USA

**Keywords:** COVID-19, cross-sectional survey

## Abstract

Computer-assisted telephone interviews (CATI) through mobile phones are a low-cost, rapid and safe way to collect data. However, decisions for how such mobile phone surveys are designed and implemented, and their data analysed, can have implications for the sample reached, and in turn affect the generalisability of sample estimates. In this practice paper, we propose a framework for extending the use of CATI–mobile phone surveys in India, which can be applied broadly to future surveys conducted using this method. Across the stages of design, implementation and analysis, we outline challenges in ensuring that the data collected through such surveys are representative and provide recommendations for reducing non-coverage and non-response errors, thereby enabling practitioners in India to use CATI–mobile phone surveys to estimate population statistics with lower bias. We support our analysis by drawing on primary data that we collected in five mobile phone surveys across nine Indian states in 2020. Our recommendations can help practitioners in India improve the representativeness of data collected through mobile phone surveys and generate more accurate estimates.

**Summary box**

- Computer-assisted telephone interviews–mobile phone surveys are susceptible to bias arising from non-coverage and non-response errors. Drawing on original data collected as part of five phone surveys across nine Indian states, we find that non-coverage and non-response errors are correlated with household income and other relevant characteristics.
- To reduce non-coverage errors, we recommend that practitioners adjust their sample estimates using poststratification weights and also discuss the limitations of this approach.
- To reduce non-response errors, we recommend that practitioners follow structured callback protocols and call households at the optimal time of the day for the given context. We found these strategies to be effective across all income groups.
- To account for non-response errors arising from households refusing to participate in the survey, we find that offering airtime incentives can improve the likelihood that households in the bottom income quartile consent to surveys, but these incentives have no statistically detectable effect on households in other income groups.

## Introduction

Household surveys are a useful tool to understand the socioeconomic conditions prevailing at a given time, which can help design better policies for the future. Given the demanding nature of face-to-face data collection and the rise in mobile phone ownership in low-and-middle-income countries (LMICs) over the last few years, researchers have begun using mobile phone surveys (MPS) to collect household survey data. Such surveys offer the promise of collecting timely, high-quality data at low costs^1 2^ in LMICs.^3–5^ MPS refers to a suite of methods, including computer-assisted telephone interviews (CATI), in which enumerators call respondents to administer surveys; Short Message Service surveys, in which data are collected from respondents using text messages; and Interactive Voice Response Surveys, in which the survey is conducted through automated voice recordings.^6^ During the COVID-19 pandemic, MPS methods have been particularly valuable to collect data required by researchers and practitioners to understand and respond to the pandemic, while maintaining physical distancing.^7 8^

**Figure 1:**
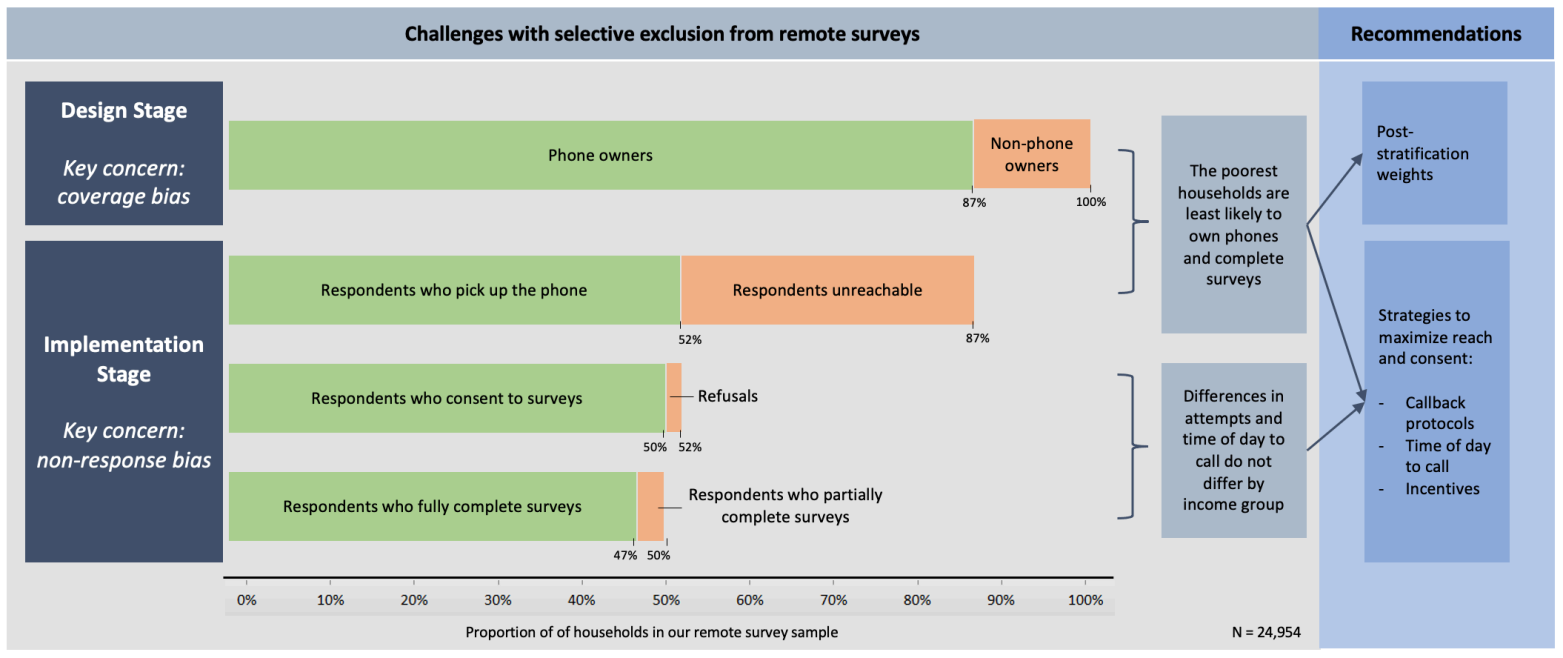
Selective exclusion from computer-assisted telephone interviews–mobile phone surveys.

We focus our analysis on CATI–MPS in India. Despite the promise of CATI–MPS in terms of cost and speed, practitioners may worry about the bias in sample estimates obtained through such surveys due to non-observation errors.^9^ These errors can arise due to differences between those who own and those who do not own mobile phones (non-coverage error),^10 11^ as well as differences between those who respond and those who do not respond to surveys (non-response error).^12^ Therefore, when implementing CATI–MPS, practitioners should pay close attention to identifying which population groups are excluded from samples and use protocols to minimise non-coverage and non-response errors. Doing so requires empirical evidence on the extent of these errors. While such evidence is growing for LMICs, to our knowledge, it is still very limited for India.^5 13^ In this paper, we propose a framework to explain the sources of bias due to non-observation errors at different stages of an MPS, design, implementation and analysis (figure 1), and recommend ways to reduce the resulting bias. To support our recommendations, we draw on primary data that we collected across five different phone surveys in nine states across India in 2020 (online supplemental box).

10.1136/bmjgh-2021-005610.supp1 Supplementary data

At the *design stage,* we recommend that practitioners document coverage bias by analysing the correlation between mobile phone ownership and socioeconomic and demographical characteristics.^14 15^ We also highlight different characteristics of sampling frames that can be used to assess their effective coverage to reduce non-coverage errors. At the *implementation stage,* we recommend protocols and interventions to decrease two important sources of non-response errors: (1) respondents who own mobile phones may not answer them, and (2) respondents who answer their mobile phone may not consent to participating in the survey. At the *analysis stage*, practitioners have a more limited toolkit and can construct poststratification weighs and/or account for mode effects.^16^ We do not discuss these methods in this paper given their sufficient treatment elsewhere.^17^ Our recommendations add to the limited body of work on CATI and MPS in the Indian context and will help practitioners to improve the quality of data collected through MPS in the future.

## Design stage

At the design stage, practitioners must decide how to construct a sampling frame of mobile phone numbers from which they can sample households. These sampling frames can be constructed from a variety of sources: through face-to-face surveys in which enumerators collect mobile numbers, through household lists maintained by the government or private companies or through random digit dialling. The sampling frames we draw on in this paper were obtained from face-to-face surveys conducted the previous year by our organisation, in which we asked households whether they possessed a mobile phone, who the primary owner was, and what the number was.

We argue that practitioners should pay attention to two features of sampling frames at the design stage: (1) what percentage of the target population is covered by the frame and how that coverage correlates with demographical and socioeconomic characteristics (‘coverage’) and (2) what percentage of mobile phone numbers are answered by the correct household (‘yield rate’).

### Coverage

CATI–MPS surveys often restrict a sampling frame to just the households in which at least one member possesses a mobile phone, especially since some of the responses may be confidential and a respondent may not feel comfortable responding on a mobile phone belonging to another household. Therefore, the metric of interest is the percentage of households where at least one member possesses a mobile phone. Practitioners should not only pay attention to the percentage of their target population that owns mobile phones, but should also investigate how mobile phone ownership correlates with relevant household characteristics. If this correlation is high, sample estimates generated from CATI–MPS are likely to suffer from greater bias. Poststratification weights could correct for observed sources of bias.^17^ However, if households in which at least one member owns a mobile phone differ from those in which none of the household members own a mobile phone along unobserved dimensions, poststratification weighting will be an imperfect solution.

In the aggregate, the coverage problem is diminishing in scope as mobile phone ownership rates increase. For example, 91.1% of rural Indian households reported owning mobile phones in 2015.^14^ The average mobile phone ownership rate in our rural districts (RD) sample is 87%, but it varies considerably across districts: ranging from 97% in Jaisalmer district, Rajasthan, to just 64% in Rayagada district, Odisha. We find that in our sample, mobile phone ownership is significantly correlated with observed characteristics like poverty: households in the bottom three income quartiles are 9.1 percentage points less likely to own mobile phones as compared with households in the top income quartile (evaluated using a two-sided t-test: p<0.01 and n=24 859; table 1). Poverty is just one dimension of exclusion. In the Indian context, mobile phone ownership can vary across gender, age and caste. Previous surveys have found that within households, women and elderly individuals are less likely to have access to mobile phones than younger men.^11^ In our sample, we find that households belonging to marginalised caste and tribal groups (officially, belonging to Scheduled Castes and Scheduled Tribes) are 7.2 percentage points less likely to own mobile phones compared with households belonging to more privileged, general caste groups (evaluated using a two-sided t-test: p<0.01 and n=24 859).

**Table 1:** Mobile phone ownership rates by income quartile*

Income quartile	Mobile phone ownership rate	N
Top 25%	93.8%	5249
50%–75%	83.9%	5795
25%–50%	85.1%	6647
Bottom 25%	85.0%	7168
Top 25%	93.8%	5249
Bottom 75%	84.7%	19 610

*Income quartile represents the range in which a household’s monthly income falls. Since our sampling frame data only included an income range as opposed to a continuous value, the number of households in each quartile is not equivalent. The bottom 25% quartile represents households whose monthly income was between 0 and 2999 Indian Rupees (INR), the middle 25%–50% quartile represents a monthly income of 3000–4999 INR, the 50%–75% quartile represents a monthly income of 5000–8999 INR, and the top 25% quartile represents a monthly income of over 9000 INR.

These findings suggest that practitioners should use poststratification weights for observed characteristics like income, caste, religion and gender, to adjust their sample estimates. However, this strategy assumes that within a particular caste–religion–income group, households that do not own mobile phones would have provided similar survey responses as households that own mobile phones. Since this assumption is tenuous, using poststratification weights may only be an imperfect solution, and practitioners may have to spend additional resources to expand the coverage of their sample, perhaps by providing mobile phones to households that do not own them during the face-to-face baseline survey, or by obtaining neighbours’ phone numbers to contact the intended household.

### Yield rate

We define yield rate to be the percentage of mobile numbers in the sample that are answered by a member of the intended household. To confirm that we reached the intended household, we matched the name of the household member who answered the phone with the list of household members we had collected during the face-to-face survey the previous year. If the name did not match, we asked the respondent the names of all other members of their household and matched these names to the list of household members.

From our experience using different sampling frames, we found that sampling frames with higher yield rates were those in which (1) mobile phone numbers were collected more recently, which increased the probability that the household retained the mobile number they had at the time of the face-to-face survey; and (2) mobile numbers were verified during the face-to-face survey, for example, by the enumerator calling the number during the face-to-face survey to confirm its accuracy.

## Implementation stage

At the implementation stage, the main source of non-observation error is non-response error, which can arise from two sources: (1) respondents may not answer the phone, and (2) those who answer the phone may not consent to participating in the survey. Practitioners should assess whether the non-response rate is correlated with relevant household characteristics and identify population groups that are less likely to respond, so that they can target efforts at reaching those groups.

In our sample, we find that both sources of non-response error are correlated with household income levels. Households belonging to the top income quartile are 7.7 percentage points more likely to answer the phone (evaluated using a two-sided t-test: p<0.01 and n=10 170; table 2) and 5.6 percentage points more likely to consent to the survey (evaluated using a two-sided t-test: p<0.01 and n=10 170; table 2), as compared with households belonging to the bottom three income quartiles. Given this difference, it is important that researchers implement protocols that improve response rates, especially among lower income households.

**Table 2:** Phone answering and consent rates by income quartile

Income quartile	Phone answering rate	Completion rate	N
Top 25%	67.5%	59.3%	2501
50%–75%	63.3%	56.7%	2180
25%–50%	62.1%	50.1%	2274
Bottom 25%	55.8%	49.9%	3215
Top 25%	67.5%	59.3%	2501
Bottom 75%	59.8%	53.6%	7669

To address concerns related to respondents not answering their phone, we recommend that practitioners use structured callback protocols and identify the best time of the day to call the respondents. To address concerns related to respondents not consenting for the survey, practitioners could consider using incentives, ensure the survey length does not exceed 30 minutes, and ensure that the enumerators’ script is conversational and engaging. Below, we discuss how callback protocols, identifying the optimal time of the day to call, and incentives can address non-response bias. We do not discuss survey length and introductory scripts given their sufficient treatment elsewhere.^1 18–20^

### How many times should you try to call a household?

Enumerators may experience challenges reaching respondents during a CATI–MPS: respondents may not answer the mobile phone, the mobile phone may be out of battery charge or airtime or outside the network coverage area. These challenges can produce biased survey estimates when they are correlated with household characteristics. In order to mitigate these challenges, enumerators will typically need to make multiple attempts to call the household before being able to speak to a member of the intended household.

We find that designing a structured callback protocol in which we specify the times during the day to call the household, and the number of attempts to make, increases the probability of reaching the intended household. In a pilot phone survey in Jharkhand, we attempted to reach 200 households without following a structured protocol. We instructed enumerators to try as best as they could to reach the intended household, but did not provide any additional guidance such as the time of the day to call them or the number of attempts to make in case previous attempts were unsuccessful. Our response rate, defined as the proportion of respondents who completed the survey, was 30%.

In a subsequent survey, we instituted structured callback protocols. We designed two protocols and sampled 100 households per protocol from the same sampling frame as the previous two pilots (Rural Districts (RD) frame, online supplemental box). In the first protocol, we randomised when during the day (morning, afternoon or evening) enumerators called the households. If the household was not reached, it was called back in the subsequent time slot (eg, if the household was initially called in the morning and did not answer, they were called back in the afternoon), up to seven times. In the second protocol, after a failed attempt, households were only called in the morning or evenings, with follow-ups placed after the hour in each slot. The response rate was 70% for the first protocol and 55% for the second protocol (the difference between the two protocols is statistically significant using a two-sided t-test: p=0.046 and n=200), with both protocols yielding higher response rates than our pilot phone surveys that lacked structured protocols. This experiment suggests that using a structured callback protocol can help practitioners improve the response rate from CATI–MPS.

**Figure 2:**
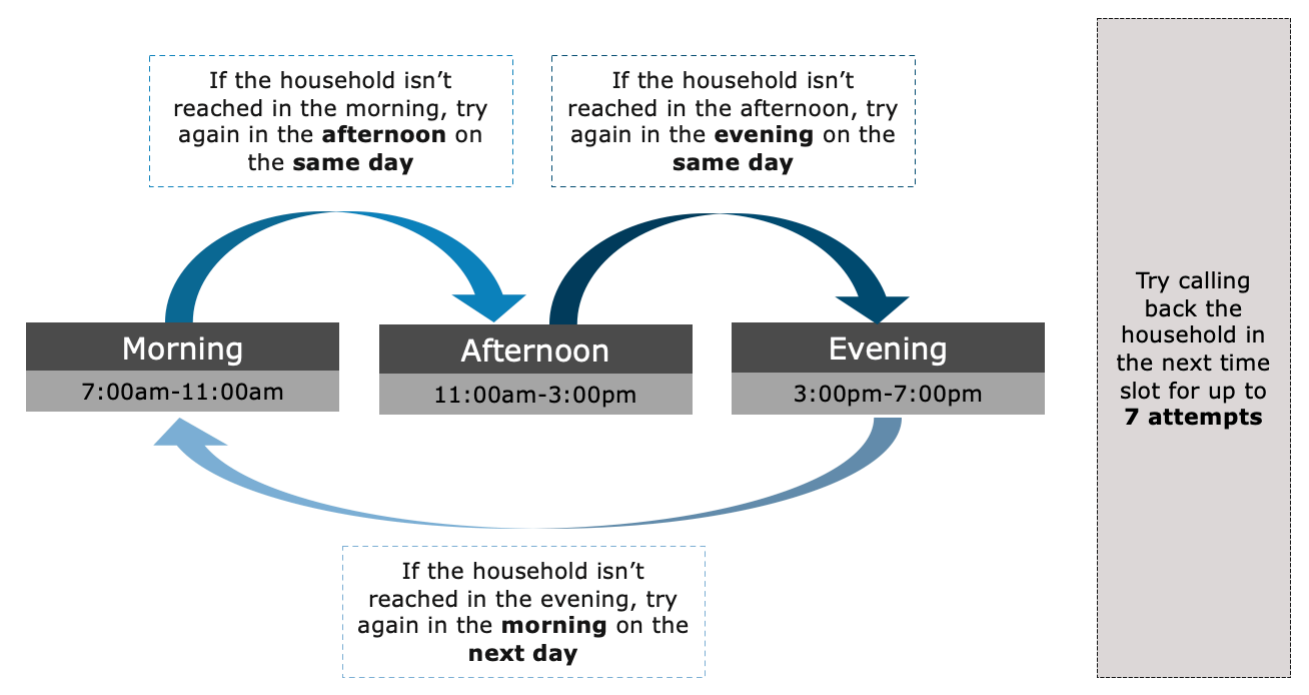
Callback protocols for computer-assisted telephone interviews–mobile phone surveys.

**Figure 3:**
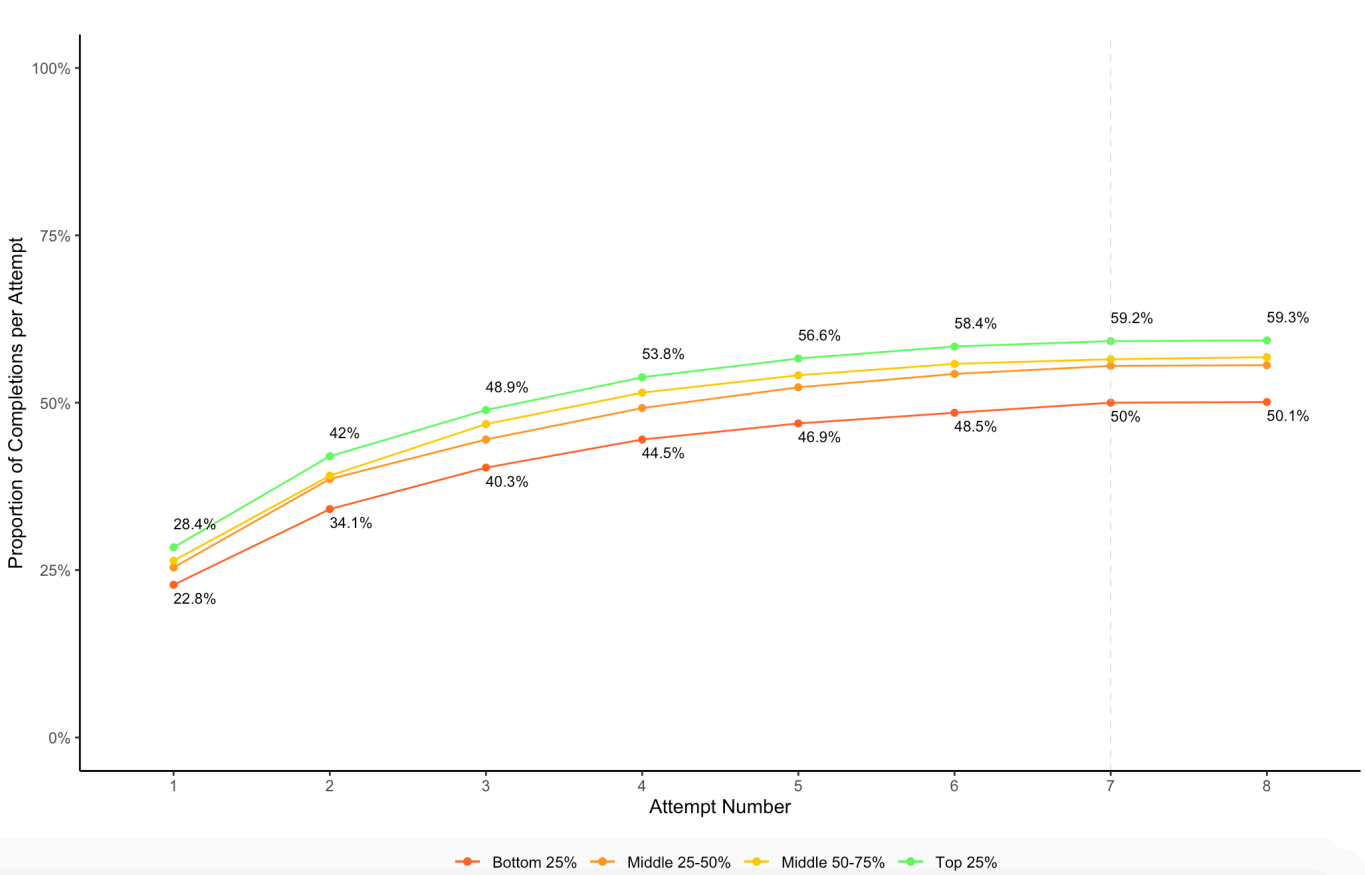
The cumulative proportion of completed surveys by attempts and income groups.

With our seven attempts protocol described in figure 2, we find that there are diminishing returns from additional attempts to reach the household. When disaggregating by income quartiles (figure 3), we observe that (1) the response rates are higher for the richest households across all seven attempts (9.2 percentage points higher for households in the top income quartile); and (2), while additional callback attempts improve the survey response rate for all income quartiles, the relative ordering of income quartiles remains the same. Therefore, we conclude that using the seven attempts protocol described in figure 2 can help improve the likelihood of reaching households across *all* income quartiles.

### When during the day should you call a household?

**Figure 4:**
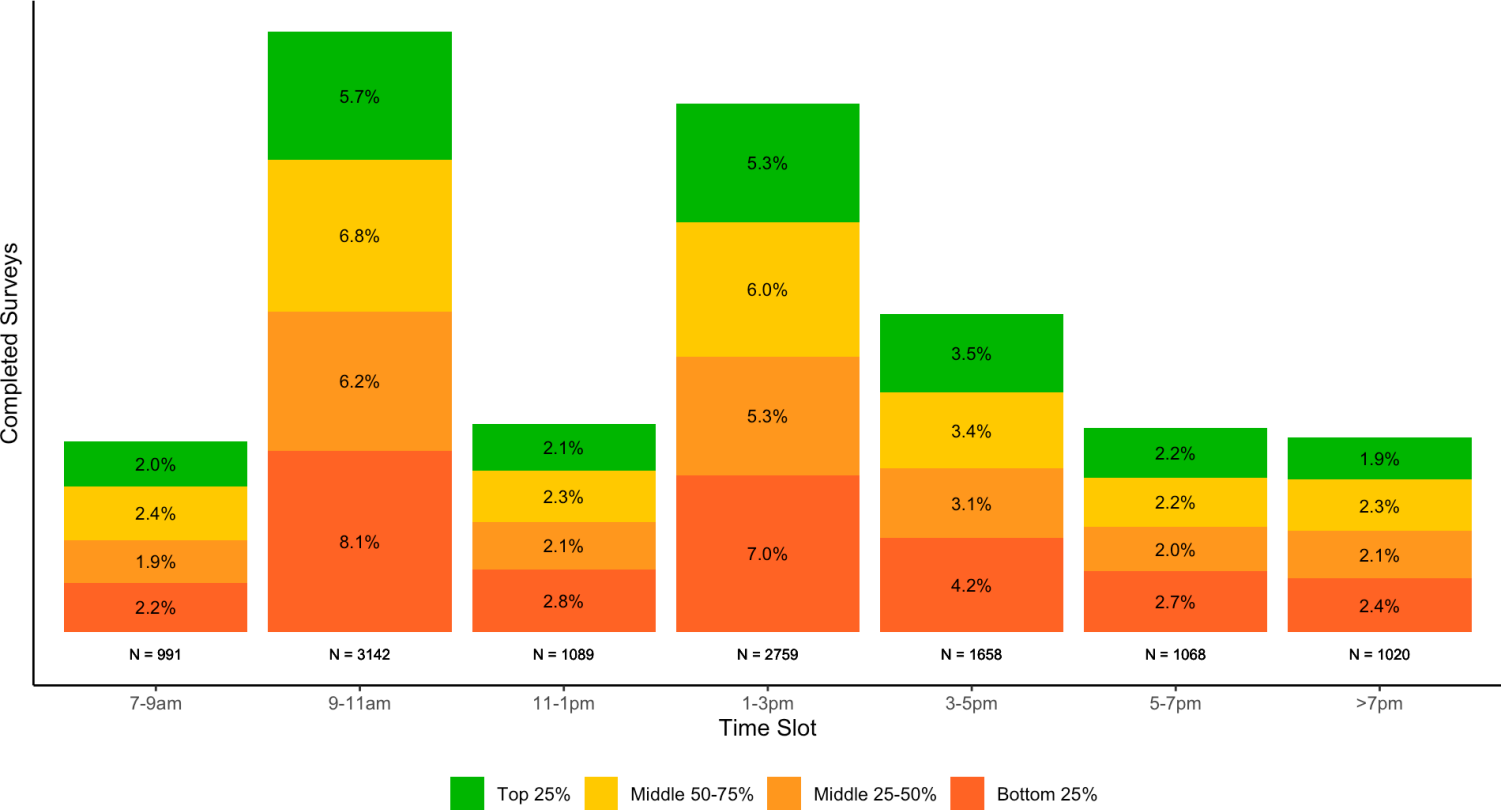
The proportion of completed surveys by time of the day and income groups.

In our surveys, we randomly assigned the time of the day when enumerators would call households for the initial call attempt. This meant that there is a roughly uniform distribution of attempts made across different times of the day. For our COVID-19 Knowledge, Attitude, and Practice (KAP) surveys, we observe that respondents were more likely to complete surveys during the morning slots. Roughly 27% of our households were reached between 9:00 and 11:00. We find that only 17.8% of households were available to be surveyed in the evenings after 17:00, likely because household members were engaged in preparing and eating dinner. Figure 4 shows the distribution of completed surveys based on the household’s income quartile and the time of the day when the household was called. The patterns are similar across income quartiles. Therefore, we conclude that the optimal time of the day to call households does not vary based on the income levels of the sampled households. However, we acknowledge that other contextual factors may affect response rates, such as seasonal variation based on the agricultural cycle. We recommend that practitioners collect data on response rates by time of the day and adjust callback protocols accordingly to improve response rates.

### Does offering incentives increase consent rates?

As outlined in figure 1, after overcoming challenges of reaching the intended households using appropriate protocols, the next step is to encourage respondents to consent to the survey. Increasing consent rates can reduce the bias arising from differences between households where a member consents or refuses to proceed with the survey after answering the call from the enumerators.

In an attempt to maximise consent rates, some researchers have hypothesised that offering incentives can increase the likelihood that respondents consent to MPS, and some studies have found that monetary incentives can be effective.^20 21^ However, since there is no physical contact between the enumerator and the respondent in CATI–MPS, there are limited options for the types of incentives that researchers can provide. One promising incentive that can be easily delivered is an airtime top-up for mobile phone users. The evidence on the effect of airtime incentives on consent rates for CATI–MPS is inconclusive.^6 22^ In our COVID-19 household surveys, we tested whether airtime incentives improved the survey completion rates among households where a member answered the phone. To do this, we conducted a two-arm individual-level randomised experiment to measure the effect of providing airtime incentives on survey completion rates for two rounds of our COVID-19 econ survey with the rural population sample (online supplemental box). In group 1, prior to seeking consent, we informed respondents that if they completed the current and a subsequent survey round, they would receive 100 INR (US$1.37) as a mobile airtime top-up after both survey rounds were completed. In group 2, we did not mention any incentives.

**Figure 5:**
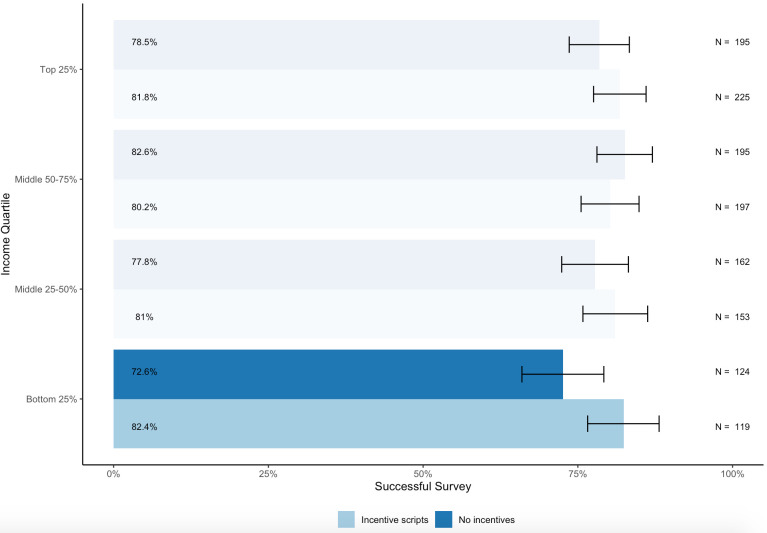
The proportion of completed surveys by incentive group and income group.

We find that the difference between completion rates, defined as the number of completed surveys of the households who answered the phone, is not statistically significant between the two groups (evaluated using a two-sided t-test: p=0.1864 and n=1370). This could be the case because most households in our sample may not value airtime incentives, particularly since Indian mobile companies offer very low rates for mobile telephony. However, when we compare the completion rates between respondents in the two treatment groups by income quartile, we observe that incentives do affect response rates for households belonging to the lowest income quartile. As shown in figure 5, for households belonging to the bottom income quartile, offering airtime incentives increased their survey completion rate by 9.8 percentage points (evaluated using a two-sided t-test: p=0.069 and n=243). The differences in completion rates across the other income quartiles are not statistically significant. This suggests that practitioners can potentially use airtime incentives to increase response rates with lower-income households. Future research is needed to examine the effectiveness of other types of incentives, such as direct cash transfers.

## Conclusion

During the COVID-19 pandemic, the use of CATI–MPS has grown substantially in India. However, these surveys remain prone to significant non-coverage and non-response errors, which could bias the resulting sample estimates. In this paper, we draw on original data from five phone surveys across nine Indian states to offer practitioners several recommendations for reducing bias at different stages of a CATI–MPS. At the design stage, we recommend that practitioners consider two features of sampling frames to minimise the non-coverage error: their coverage (proportion of households where at least one member possesses a mobile phone) and the yield rate (percentage of mobile numbers in the sample that are answered by the correct household). If the coverage of the sampling frame is correlated with relevant household characteristics, practitioners can use poststratification weights, but this strategy is an imperfect solution. At the implementation stage, we recommend various steps to reduce non-response rates. First, using a structured callback protocol can improve response rates across households of all income quartiles. Second, calling at different times of the day can help practitioners reach households of all income groups, with morning slots yielding higher response rates across all groups. Third, providing airtime incentives in return for completing the survey improves the response rates among the poorest households. These findings and recommendations can help practitioners reduce bias at each stage of the survey and generate more accurate sample estimates from CATI–MPS.

## Data Availability

Data are available upon request.
