# Supplementary material for: Who do phone surveys miss, and how to reduce exclusion: recommendations from phone surveys in nine Indian states
Source: BMJ Glob Health. 2021 Aug 11;6(Suppl 5):e005610. doi: 10.1136/bmjgh-2021-005610 (PMC8359516; doi:10.1136/bmjgh-2021-005610)
Supplement: Supplementary data [file bmjgh-2021-005610supp001.pdf]

SUPPLEMENTARY BOX: DATA SOURCES

In our CATI mobile phone surveys conducted between January and September 2020, we surveyed respondents for whom we had collected mobile phone numbers through different face-to-face surveys during 2019. Details of the sampling frames used are referenced throughout the paper by the acronyms listed below:

1. **Rural Districts Survey (RD)**

   Description: A sample of 1,000 households per district, representative of the district's adult population, randomly sampled using publicly available electoral rolls.
   States: Assam, Bihar, Jharkhand, Madhya Pradesh, Maharashtra, Odisha, Rajasthan, Uttar Pradesh
   Baseline Timeframe: September-November 2019
   Survey topics: COVID-19 knowledge, attitudes, and practices, economic impacts of COVID-19 lockdown, migration, consumption, food security
   Survey duration: 25 minutes

2. **Rural Populations Survey (RPS)**

   Description: This sample was collected by another organization on behalf of the National Rural Livelihoods Program. The sample is representative of Self-Help Group members at the state level.
   States: Madhya Pradesh, Uttar Pradesh, Bihar
   Baseline Timeframe: October - December 2019
   Survey topics: Migration, Consumption, Labor, Agriculture, Relief, Health
   Survey duration: 30 minutes

We use data from five CATI mobile phone surveys and one IVR survey completed between January and September 2020. We introduced different experiments in each survey, and across all surveys, we collected data on the households reached and not reached. Details of these surveys are highlighted below:

Pilot survey

Description: We attempted to call 200 households in Jharkhand without a structured callback protocol. These households were sampled from the Rural Districts sampling frame.

IVRS Callback Protocol Experiment

Description: We attempted to call 200 households using interactive voice response survey (IVRS) technology in Jharkhand. These households were sampled from the Rural Districts sampling frame.

COVID KAP (Rounds 1 and 2)

Description: We attempted to call 11,000 households per round to ask questions about COVID-19 knowledge, attitudes and practices. These households were sampled from the Rural Districts sampling frame.

COVID Econ (Rounds 1, 2, and 3)

Description: We attempted to call between 8,300 and 10,800 households, depending on the survey round, to ask questions about the impact of COVID-19 on migration, agriculture, consumption, and relief. These households were sampled from the Rural Population Survey sampling frame.
